# Global Research on Quality of Life of Patients with HIV/AIDS: Is It Socio-Culturally Addressed? (GAP_RESEARCH_)

**DOI:** 10.3390/ijerph17062127

**Published:** 2020-03-23

**Authors:** Giang Thu Vu, Bach Xuan Tran, Chi Linh Hoang, Brian J. Hall, Hai Thanh Phan, Giang Hai Ha, Carl A. Latkin, Cyrus S.H. Ho, Roger C.M. Ho

**Affiliations:** 1 Center of Excellence in Evidence-Based Medicine, Nguyen Tat Thanh University, Ho Chi Minh City 700000, Vietnam; giang.coentt@gmail.com; 2 Institute for Preventive Medicine and Public Health, Hanoi Medical University, Hanoi 100000, Vietnam; bach.ipmph@gmail.com; 3 Bloomberg School of Public Health, Johns Hopkins University, Baltimore, MD 21205, USA; brianhall@umac.mo (B.J.H.); carl.latkin@jhu.edu (C.A.L.); 4 Center of Excellence in Behavioral Medicine, Nguyen Tat Thanh University, Ho Chi Minh City 700000, Vietnam; chi.coentt@gmail.com (C.L.H.);; 5 Global and Community Mental Health Research Group, University of Macau, Macau 999078, China; 6 Institute for Global Health Innovations, Duy Tan University, Da Nang 550000, Vietnam; haipt.ighi@gmail.com; 7 Department of Psychological Medicine, National University Hospital, Singapore 119074, Singapore; cyrushosh@gmail.com; 8 Department of Psychological Medicine, Yong Loo Lin School of Medicine, National University of Singapore, Singapore 119228, Singapore; 9 Institute for Health Innovation and Technology (iHealthtech), National University of Singapore, Singapore 119077, Singapore

**Keywords:** scientometrics, HIV/AIDS, bibliometric, quality of life

## Abstract

Quality of life (QOL) has been considered as an important outcome indicator in holistic care for HIV-infected people, especially as HIV/AIDS transforms from a fatal illness to a chronic condition. This study aimed to identify trends and emerging topics among research concerning the QOL of people living with HIV/AIDS (PLWHA). The analyzed data were English papers published from 1996 to 2017, searched and extracted from the Web of Science Core Collection. Collaborations between countries and the correlation between the keywords were visualized by VOSviewer while the abstracts’ content was analyzed using exploratory factor analysis and Jaccard’s’ similarity index. There has been an increase in both the number of publications and citations. The United Nations of America leads in terms of paper volume. The cross-nation collaborations are mainly regional. Despite a rather comprehensive coverage of topics relating to QOL in PLWHA, there has evidently been a lack of studies focusing on socio-cultural factors and their impacts on the QOL of those who are HIV-infected. Further studies should consider investigating the role of socio-cultural factors, especially where long-term treatment is involved. Policy-level decisions are recommended to be made based on the consideration of cultural factors, while collaborations between developed and developing nations, in particular in HIV/AIDS-ridden countries, are strongly recommended.

## 1. Introduction

Human immunodeficiency viruses (HIV) is one of the leading causes of disability and mortality worldwide, with more than 76.1 infected people and 35.0 million deaths [1,2,3]. In 2017, there were 1.8 million people newly infected with HIV and Acquired immunodeficiency syndrome (AIDS), 36.9 million people living with HIV and AIDS (PLWHA), and 940,000 deaths related to life-threatening infections and cancers [4]. Hence, ensuring sufficient care and treatment, as well as treatment provision, has become a challenge for global public health systems.

Quality of life (QOL), as noted by the existing literature, has been described as an umbrella term for a variety of human needs, including the position in life, goals, standards, expectations, and concerns in the context of the culture and value systems. It manifests within patients as symptomatic, social functioning, and spirituality [5]. In terms of health promotion, health-related quality of life (HRQOL) is also considered as a priority health indicator. Since 1996, optimizing adherence to Antiretroviral Therapy (ART) has brought the chance to transform HIV—an incurable disease—into a chronic health condition [6], which in turn prolongs the life of PLWHA and improves their QOL [6]. In many settings, poor QOL is associated with a lower immune response, non-adherence, poor mental health, and greater disease severity [7,8,9]. Therefore, QOL attracted great attention from regulatory authorities and health providers as an important outcome to evaluate the effectiveness of HIV treatment [10,11,12,13]. People who were effectively treated with HAART, however, have been found to have a lower QOL compared with other long-term chronic illnesses [14]. Over time, the expansion of HAART coverage not only prolonged the life expectancy of PLWHA, but also boosted the innovation of new QOL instruments to adapt to the complexity of care. The previous literature has reported a high burden of comorbidities suffered by PLWHA, as well as adverse side impacts of long-term treatment on their health [15,16], while a complex combination of psychological and social factors which also influence their physical, mental and social conditions, directly and indirectly, affect their QOL [17,18,19,20,21,22]. On the other hand, in recent years, the availability of early HIV diagnosis, antiretroviral (ARV) treatments and enhanced healthcare services have been found to support the improvement in the QOL of PLWHA [23]. 

Thus, in order to improve the quality and effectiveness of HIV/AIDS treatment and prevention programs, qualitative as well as quantitative analyses on the QOL of PLWHA are needed. In 2017, Cooper, et al. conducted a systematic review on the finding of existing reviews on QOL of the HIV/AIDS-infected population in various aspects, including the development, validation, and effectiveness of the most commonly used instruments [12]. Though informative, reviews of such kind suffer from the limitation of a narrow focus on specific questions or issues. In order to broaden the scope of research, a new approach is needed that has the ability to cover a large volume of global data on QOL in PWLHA research and allows for complex analysis to identify the research trend [12,18,24,25].

Our study adopts the scientometrics approach that gathers and analyze publications on a global level, coupled with more a technical analysis approach applied to the content of papers’ title and abstract to identify emerging research topics as well as the current level of international collaboration in research on the QOL of PLWHA. This study aims to supplement the current literature while uncovering research gaps, suggest directions for future studies, and act as a reference point for priority settings and strategies initiating in HIV/AIDS management. 

## 2. Materials and Methods 

### 2.1. Study Design

Serving as the best approach to evaluate evidence, the increase in the number of systematic reviews of QOL regarding HIV/AIDS could provide an insightful view of various aspects, including evaluating the effectiveness of instruments, clinical intervention, and HIV/AIDS programs in vulnerable populations. In addition to having a limited scope of research; such reviews also remain lack of the comparison of findings over time and overlap with information. Several researchers use bibliometrics with the expectation that it could fill the gap in the literature and provide the research trend using quantitative analysis. It can be seen that the bibliometric approach sometimes could not draw a full picture because the majority only present the number without a comprehensive reading of the literature [26]. In order to provide a comprehensive view of the current status of the quality of life in terms of the HIV/AIDS literature, we conducted the scientometric approach combined with content analysis. 

The current study is a part of a larger project, Global Analysis for Policy in Research (GAPRESEARCH), that aims to set priorities in global health evolution and provide empirical evidence for designing effective interventions and policies [27,28,29,30,31,32]. The findings of this study, therefore, can be used as a reference point for directing investments, allocating resources, and crafting policies.

### 2.2. Search Strategy

A search for HIV/AIDS publications was performed on the Web of Science (WoS) Core Collection. This database was prioritized because it has covered all scientific publications with full cited reference indexing since 1900 and allowed downloading information with a diversity of research disciplines that far outweigh other databases such as Scopus or MEDLINE [32]. 

**Figure A1:**
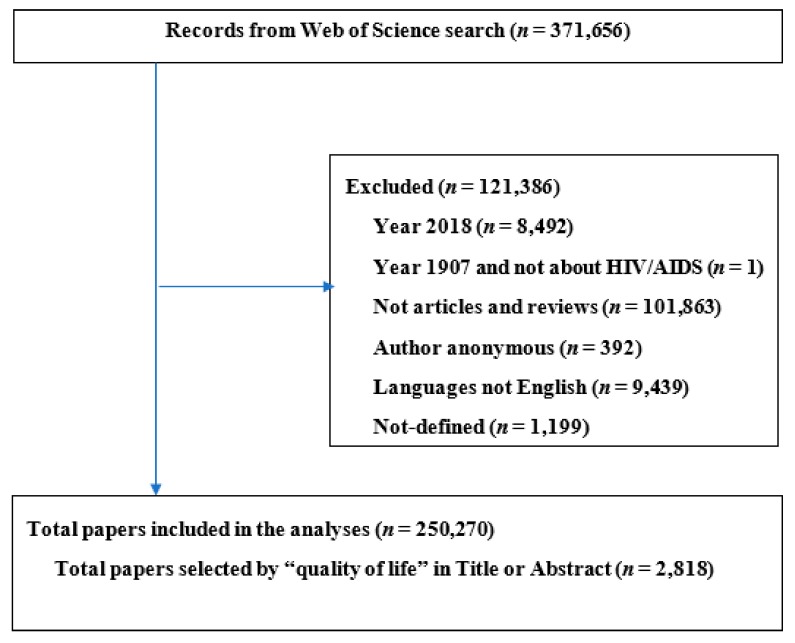
Selection of papers. The searching and paper selection process identified 250,270 related to HIV/AIDS. Among those, 2818 papers included the terms “quality of life”.

We applied a search query containing the search terms of “HIV” OR “human-immunodeficiency-virus” OR “AIDS” OR “Acquired-Immune-Deficiency-Syndrome”. Data were extracted in a unit of 500 publications from the first publication in the dataset end up in 2017. As the process was conducted in July 2018, we excluded all papers published in 2018 as the partial coverage of publications published in 2018 would not fully reflect the publication trend of the year. The selection criteria for study subjects were English peer-review articles, including original articles and reviews (see Figure A1). Any paper with anonymous or no authors would be excluded. In the next step, we continue refined relevant research by using the research term “quality of life”, articles could be removed if they did not include “quality of life” in titles and abstracts. Any disagreement during the screening process could be discussed with a senior researcher. 

### 2.3. Summary Measures

The retrieved data consisting mainly of publication indexes were contained the following information: Title; years of publication; the total number of papers; citations up to 2017; usages/number of downloads; keywords (authors’ keywords); authors’ affiliations; most prolific countries and collaborations. In order to describe the change of publication over time, we calculated several fundamental domains including the speed of publication (total number of papers), the level of reader attention (mean cited rate per year), the level of short-term and long-term interest (mean usage rate last 6 months/5 years). We also illustrated the major topic clusters and landscapes of QOL in the field of HIV/AIDS by calculating the frequency of co-occurrence of keywords and synthesized from abstract’s content. 

### 2.4. Data Analysis

The extracted data were sorted by Macro in Microsoft Excel to calculate the indexes. The connection among countries by sharing co-authorships’ data (we applied full counting for papers sharing by more than one country), networks of co-occurrence authors’ keywords, and clusters of topic groups were visualized by VOSviewer (version 1.6.11, Center for Science and Technology, Leiden University, the Netherlands). The cluster topics of QO were then identified from the frequency of keywords and named by expert opinions. 

The exploratory factor analysis (EFA) and Jaccard’s similarity index were performed using STATA software version 15.0. This index was defined as the magnitude of the intersection divided by the magnitude of the union of two sets of co-occurring terms; thus, multi-dimensional scaling could be used to adjust a point for a topic category, the distance between items and color presented the partnership of certain key terms. To measure the likelihood of research trends (e.g., emerging research domains and landscapes), we utilized exploratory factor analysis (EFA), which allows us to test the variance in the domains and landscape appearing from the abstract’s contents. The summary of the technique used for analyzing is described in Table A1. 

## 3. Results

Table 1 shows an expansion in the volume of publications on QOL among HIV/AIDS populations. The period between 2007 and 2017 saw the number of papers grow from 114 to 234. In particular, the total citation (from the year published up to 2017) has also risen remarkably in 2005 and 2013. 

Table 2 indicates the frequency of countries by counting such study settings in the mentioned abstracts. In the top 10, except countries with the highest HIV prevalence, such as South Africa, the number of studies were mostly produced in upper-middle-income and high-income countries (United States, Brazil, China, Canada, Thailand, England, Australia) [33]. The low and middle-income countries such as Botswana, Zambia, Lesotho, Swaziland had below 10 papers, even though these countries have been reported as having the highest rate of HIV/AIDS [34].

**Figure 1:**
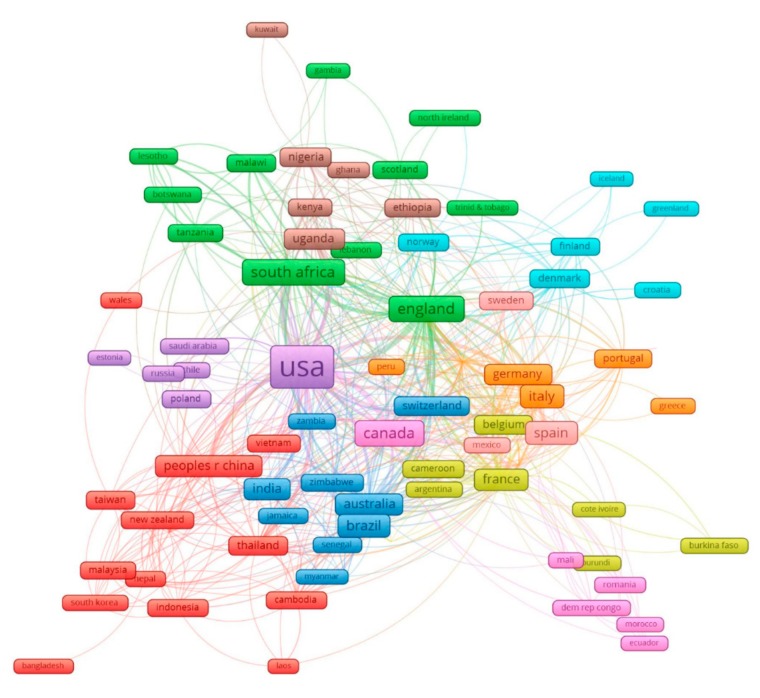
The network of 102 countries having international co-authorships in quality of life research in HIV/AIDS.

Figure 1 displays the global network between 102 countries having co-authorships of selected papers. These countries have been classified into 10 clusters of at least five countries, depending on their level of international collaboration. 

Cluster 1 (red) refers to the network of countries in the two regions: South-East Asia and the Western Pacific. Cluster 2 (purple) indicates the link of the USA with European countries and Saudi Arabia. Cluster 3 (blue) illustrates international collaborations between the Americas, South Africa, and some countries in Asia. Cluster 4 (brown and green) demonstrates the co-authorship between countries within Southeast Africa. In addition, they have an additional link with England. The European countries tend to associate with each other by geographical areas, such as orange, pink, light blue, and yellow (French West Africa). 

**Figure 2:**
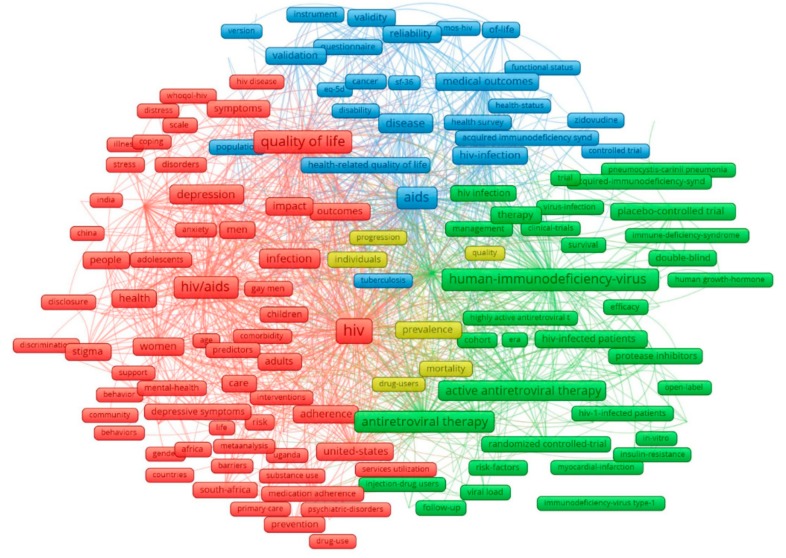
The most common author keywords. Note: the principal components of the data structure were visualized regarding the colors of the nodes; the node size was identified by the keywords’ occurrences; the closer the nodes are, the stronger the association between two keywords are.

Figure 2 describes the core components of the keywords with the most common groups of terms. There were four major clusters that emerged from 205 most frequent co-occurrence keywords with a minimum frequency of 20 times. Three major clusters (red, blue, and green) indicate three topics of quality of life. 

Cluster 1 (red) covers mental health, social, and associated factors. It includes psychological disorders (depression, anxiety, stress); social behavior (stigma, discrimination); specific HIV groups (such as gay, children, women, and men), and regions (India, South Africa, Uganda). Cluster 2 (blue) presents physical health-related aspects: treatment, outcome, mortality, and disability. Cluster 3 (green) indicates ART adherence studies: study design and laboratory results.

The top 50 emerging research domains have been discovered from the content analysis of abstracts using exploratory factor analysis (Table 3). Mental health (62.6% of papers containing mental health-related keywords) appears to receive more attention from researchers compared to other health problems (e.g., chronic conditions 23.4%, or cost-effectiveness 37.5%). Other major domains cover coping strategies and social support. Nearly half of the articles have keywords related to the randomized and controlled trial. Meanwhile, the keywords concern comorbidities account for less than 10%.

**Figure 3:**
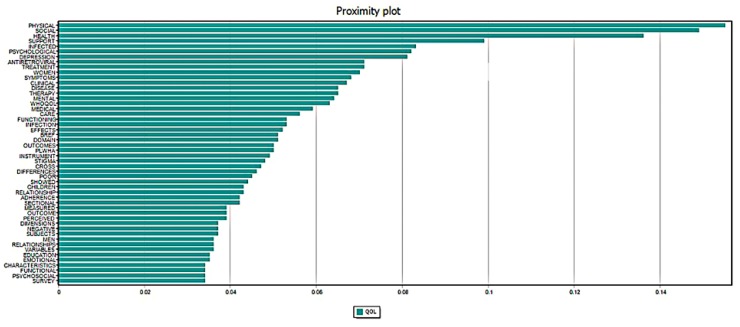
Proximity Plots of “quality of life” with the top 50 most frequent concurrence terms in abstracts.

Figure 3 illustrates the similarity between QOL and the top 50 co-occurrence terms. In particular, physical, social, and health were the most common terms that co-occurred with QOL in all abstracts. The term related to women was more common than that of men in the literature. Antiretroviral, treatment, and infection were also co-occurrence terms with a high frequency of appearance. 

## 4. Discussion

By analyzing the volume and abstract contents of global publications on the QOL of PLWHA during 1996–2017, our research captured and visualized the level of attention, current research trends, and the global networking of researches. The results show rather extensive coverage of topics in the existing literature, ranging from physical-related aspects to mental health, from issues concerning clinical trials to social support. Nonetheless, the current bibliography shows the lack of socio-cultural factors involved in the development and measurement of QOL. 

Our study reports an increase in the number of papers using QOL as an important instrument for evaluating HIV/AIDS interventions since 1996, when highly active antiretroviral therapy (HAART) was first introduced [6]. This result is in line with a study conducted by Eltony et al., which confirmed an increasing trend in the volume of publications on QOL of PLWHA [35]. On the other hand, our findings draw a troubling picture regarding the degree of inequality in contributions and collaborative partnerships across settings. While most HIV incidence is located in LMICs, for instance, nearly 70% of individuals infected with HIV live in Sub-Saharan Africa [36], the highest amount of relevant studies belonged to high-income countries. It is widely acknowledged that the combination of low socioeconomic conditions and limited access to health services result in increasing HIV prevalence in sub-Saharan Africa, the Caribbean, and Central Asia [37]. Therefore, these countries may be more focused on the prevention of HIV transmission rather than investing interventions to improve mental health or social situation, for instance, to reduce stigma against HIV patients when accessing general health facilities. The reduction in global funds for HIV—the major source of financing for HIV/AIDS management in LMICs—would also lead to a lack of funding for the crucial activities of collecting empirical data, planning essential investigations, and HIV/AIDS management strategies [38]. Meanwhile, cross-regional collaborations, and especially research partnerships between high-income countries and their low-middle-income counterparts, have been found to still be rather limited (Figure 1). These findings call for more collaboration efforts between developed and developing nations, in which support both in terms of finance and knowledge/ technology should be transferred from advanced to disadvantaged regions. In addition, further research in favor of economic evaluation should be conducted to identify the appropriate interventions in the context of limited funding for HIV/AIDS management. 

Knowing the association between the QOL of PLWHA and the effectiveness of HIV programs, QOL has been used as a criteria in assessing HIV/AIDS prevention programs, clinical treatment, and harm reduction strategies [39,40,41]. This is reflected in the finding of our study, as terms relating to QOL measurements like MOS-HIV, EQ-5D, SF-36, and WHOQOL-HIV are found to frequently co-occur with QOL in analyzed publications (Figure 2), while the EFA of abstract content identifies QOL measurements to be an emerging research domain (Table 3). EQ-5D and SF-36 have been broadly used thanks to their ability to be adopted for economic analyses. Meanwhile, MOS-HIV and WHOQOL-HIV have been developed and validated as QOL measurements specifically for the HIV/AIDS-infected population [42,43,44,45,46].

Even with the advancement of health services as well as the high ART coverage, the HIV/AIDS programs remain complex, contextual, and are often referred to as complicated because appropriate recommendations vary according to subpopulation and epidemiological context [23]. Previous studies have reported that in LMIC, a combination of factors, instead only one or two major ones, have major impacts on optimal adherence and rates of virological suppression when a patient is lost to follow-up [23]. The combined language and ethnicity profile of a country has been found to significantly influence culturally sensitive healthcare services—those at risk of HIV infection may face delayed treatment initiation and access to prevention services in regions where stigma against infectious diseases is common, for instance [47]. Some of the most HIV/AIDS-ridden nations in North-Central Africa, such as Cameroon, Nigeria, and the Democratic Republic of the Congo, have been found to also be the most culturally diverse countries [48]. The absence of culture-related terms like language, belief and religion in the keywords and abstract content of our analyzed publications suggest a gap in the research concerning the QoL of PLWHA. Further studies thus may consider assessing the role of cultural factors on QoL of PLWHA, as well as the impacts of diverse beliefs, for instance, on the effectiveness of the programs initiated to improve the QoL of those infected with HIV. Similarly, those involved in developing HIV/AIDS management programs, including policy-makers and non-governmental organizations, should take into account the impact of cultural factors. 

The analysis of a principal component of terms in titles and abstracts reveals that QOL tends to co-occur with terms relating to mental disorders and high-risk populations, including adolescents, children, women, and gay. Disclosure, discrimination, and stigma have also been found to appear together with the aforementioned terms, along with keywords like barriers and primary care (Figure 2). This finding suggests that topics on the mental health consequences of HIV infection and its treatments, barriers to treatment due to stigmatization and social-related issues like reluctance to disclosure have been covered in the existing literature. The focus on single domains of QOL, such as physical [14,49,50], psychological [49,51,52,53], social [50,52,53,54], and environmental [55,56] can be said to be common in research concerning QOL among PLWHA. However, given the complex, multi-dimensional nature of the QOL construct, the lack of contextualized factors (sociological perspective, culture, religion for instance) in the titles and abstracts of published papers, as our results reveal, can undermine the power and scope of impact of QOL on PLWHA and the effectiveness of treatments. Therefore, further studies are encouraged to address more contextualized factors and consider adopting multiple QOL measures when attempting to evaluate the association and influence of QOL on PLWHA. 

Despite the positive findings of the study, several limitations should be mentioned. As our search strategy was conducted via Web of Science Core Collection solely and only English reviews and articles were included, despite the extensive coverage of WoS and the dominance of English publications, there is a chance that relevant publications not recorded in such a database and/or in other languages would be missed. Our decision to use only the term “quality of life” when conducting a publication search would also filter out possibly related papers where variations of the construct such as “satisfaction with life”, “well-being”; “satisfaction” or “value of life” were used instead. Thus, further researches are strongly encouraged to consider investigating deviations of “quality of life” both as a term and a concept, especially research with a sociological focus. In addition, future studies may also be conducted in the form of systematic reviews and meta-analyses, for instance, on how QOL can or has been used as a measurement for assessing the effectiveness of HIV/AIDS treatments or interventions.

## 5. Conclusions

Using bibliometrics analysis, we illustrate the development and current global trends of research on the QOL of PLWHA. Meanwhile, the results of the text mining techniques adopted provide a picture of current emerging research trends and topics and highlight research gaps. Despite a rather comprehensive coverage of topics relating to QOL in PLWHA, there has evidently been a lack of studies focusing on socio-cultural factors and their impacts on the QOL of those that are HIV-infected. Further studies should consider investigating the role of socio-cultural factors, especially where long-term treatment is involved. Policy-level decisions are recommended to be made based on the consideration of cultural factors, while collaborations between developed and developing nations, in particular with HIV/AIDS-ridden countries, are strongly recommended. 

## Figures and Tables

**Table 1:** General indicators of publications

Year Published	Total Number of Papers	Total Citations	Mean Cite Rate per Year ^1^	Total Usage* Last 6 Months	Total Usage* Last 5 Years	Mean Use Rate Last 6 Months ^2^	Mean Use Rate Last 5 Years ^3^
2017	234	11,965	51.1	356	1,151	1.5	1.0
2016	215	11,221	26.1	211	1,619	1.0	1.5
2015	218	10,985	16.8	174	1,972	0.8	1.8
2014	211	9,638	11.4	107	1,891	0.5	1.8
2013	213	9,866	9.3	96	2,286	0.5	2.1
2012	185	8,773	7.9	52	1,692	0.3	1.8
2011	169	8,476	7.2	65	1,315	0.4	1.6
2010	167	7,462	5.6	46	1,160	0.3	1.4
2009	140	6,445	5.1	62	908	0.4	1.3
2008	122	5,468	4.5	53	818	0.4	1.3
2007	114	5,639	4.5	33	622	0.3	1.1
2006	96	4,588	4.0	41	606	0.4	1.3
2005	112	5,265	3.6	61	767	0.5	1.4
2004	80	3,746	3.3	36	414	0.4	1.0
2003	73	3,012	2.8	25	374	0.3	1.0
2002	58	2,579	2.8	10	226	0.2	0.8
2001	51	2,163	2.5	11	264	0.2	1.0
2000	66	2,759	2.3	11	309	0.2	0.9
1999	49	2,291	2.5	13	212	0.3	0.9
1998	56	2,385	2.1	16	214	0.3	0.8
1997	39	1,708	2.1	7	128	0.2	0.7
1996	44	1,455	1.5	11	169	0.3	0.8

* Usage: downloaded time; ^1^ Mean cited rate per year = Total citations/(Total citations × (2018-that year)); ^2^ Mean usage rate last 6 months = Total usage last 6 months/Total number of papers; ^3^ Mean use rate last 5 years = total usage last 5 years/(total number of papers × 5).

**Table 2:** A number of papers by countries as study settings.

No.	Country Settings	Frequency	%	No.	Country Settings	Frequency	%
1	United States	271	21.1%	31	Sweden	8	0.6%
2	South Africa	108	8.4%	32	Zambia	8	0.6%
3	India	74	5.8%	33	Cambodia	7	0.5%
4	Brazil	60	4.7%	34	Ghana	7	0.5%
5	China	60	4.7%	35	Lesotho	7	0.5%
6	Uganda	52	4.0%	36	Netherlands	7	0.5%
7	Canada	42	3.3%	37	Swaziland	7	0.5%
8	Thailand	32	2.5%	38	Switzerland	7	0.5%
9	United Kingdom	30	2.3%	39	Hong Kong	6	0.5%
10	Australia	29	2.3%	40	Jamaica	6	0.5%
11	Ethiopia	27	2.1%	41	Japan	6	0.5%
12	Niger	25	1.9%	42	Nepal	6	0.5%
13	Nigeria	25	1.9%	43	Germany	5	0.4%
14	Malawi	21	1.6%	44	Mexico	5	0.4%
15	Viet Nam	20	1.6%	45	Peru	5	0.4%
16	Ireland	19	1.5%	46	Wallis and Futuna	5	0.4%
17	Taiwan	18	1.4%	47	Colombia	4	0.3%
18	Rwanda	17	1.3%	48	Georgia	4	0.3%
19	Kenya	15	1.2%	49	Haiti	4	0.3%
20	Iran	14	1.1%	50	Jersey	4	0.3%
21	Spain	14	1.1%	51	Portugal	4	0.3%
22	Tanzania	14	1.1%	52	Romania	4	0.3%
23	Zimbabwe	14	1.1%	53	Singapore	4	0.3%
24	Malaysia	13	1.0%	54	Burkina Faso	3	0.2%
25	Cameroon	12	0.9%	55	Democratic Republic of the Congo	3	0.2%
26	Italy	12	0.9%	56	Republic of the Congo	3	0.2%
27	France	11	0.9%	57	Finland	3	0.2%
28	Botswana	9	0.7%	58	Guinea	3	0.2%
29	Indonesia	9	0.7%	59	Lebanon	3	0.2%
30	Puerto Rico	8	0.6%	60	Mali	3	0.2%

**Table 3:** Top 50 research domains emerged from exploratory factor analysis of all abstracts’ contents.

Name	Keywords	Eigen Value	FREQ	% Cases
Mental health summary	Medical Outcomes Study (MOS); summary; mental; survey; physical; scores; medical; score; health related quality of life (HRQOL); outcomes; short form (SF)	26.1	4123	62.60%
Criteria; controlled trials	Criteria; trials; objectives; controlled; main; evidence; performed; review; evaluate	1.9	2357	48.90%
The World Health Organization Quality of Life brief (WHOQOL-BREF); Domain	WHOQOL; BREF; Domain; domains; world; version; QOL; psychological; social	3.3	2335	44.20%
Randomized; Controlled trial	Randomized; trial; placebo; controlled; weeks; week; groups; primary; intervention	7.2	2312	39.30%
Cost-effectiveness; costs	Cost; costs; effectiveness; effective; economic; model; year	1.9	1643	37.50%
Coping strategies; social support	Coping; strategies; support; social	1.3	1522	37.10%
Depressive; anxiety and depression	Depressive; depression; symptoms; anxiety; psychiatric; psychological	2.7	1722	36.10%
Reliability and validity; item scale	Validity; reliability; item; items; instrument; factor; scales; measure; good; scale	2.2	1923	36.10%
Care access	Access; services; care	1.3	1220	33.40%
Role functioning	Role; function; cognitive; functioning	1.5	1025	27.30%
Viral load; count	Load; viral; count; counts; cells	1.8	1384	27.00%
Literature; review	Literature; review; evidence	5	919	24.10%
Sex; men and women	Sexual; men; sex; women	1.8	1002	23.90%
Chronic conditions	Conditions; chronic; diseases	1.6	840	23.40%
Stigma; disclosure	Stigma; disclosure; perceived; negative	3.7	837	23.20%
Cross-sectional	Sectional; cross; prevalence	2.5	1128	23.00%
Side effects	Side; effects	1.5	815	22.80%
Follow-up period	Period; follow; month; year	2.3	852	22.50%
Body mass; fat loss	Mass; body; fat; weight; kg; loss; nutritional; exercise; testosterone; index	3.3	1169	21.40%
Drug users	Users; methadone; drug; substance	2.3	766	21.00%
Outcome measures	Outcome; measures	1.3	718	20.60%
Control	Controls; control; intervention	1.7	712	20.10%
Anti; development	Anti; development; resistance; therapeutic	1.2	712	19.70%
Palliative; cancer pain	Palliative; cancer; advanced; pain	1.9	620	18.20%
Adherence to medication	Medication; adherence	1.6	639	18.10%
Developed countries	Countries; developed; settings	1.2	600	18.00%
Screening; early	Screening; early; settings	1.2	500	15.50%
Combination	Combination; response	1.3	416	13.50%
Demographic	Demographic; characteristics	1.5	456	13.40%
Children	Children; adoslescents; caregivers; family	1.7	499	13.30%
EuroQOL (EQ); HRQOL demension	EQ; HRQOL; Dimensions	1.6	445	13.30%
Reduction	Reduction; improvement	1.4	407	13.10%
Morbidity and mortality	Mortality; morbidity	1.7	497	12.60%
Inhibitor; protease inhibitor (PI) regimens	Inhibitor; PI; regimen; regimens	3.1	498	12.20%
Death	Death; hospital	1.3	357	12.00%
Emotional	Emotional; functional	1.3	358	11.80%
Exercise	Exercise; activity; week	1.3	374	11.70%
Moderate to severe	Severe; moderate; haemophilia	1.5	390	11.70%
Long-term	Term; long	2.1	522	11.20%
Adverse effect	Events; adverse	1.4	426	11.20%
Cytomegalovirus (CMV); Prophylaxis	CMV; prophylaxis; infections	1.4	348	10.80%
South Africa	South; Africa	2	344	8.60%
Food	Food; people living with HIV (PLHIV); nutritional	1.3	225	6.90%
Facial	Facial; satisfaction	1.3	177	5.90%
Hepatitis	Hepatitis; hepatitis C virus (HCV)	1.9	215	5.60%
Sleep	Sleep; fatigue	1.4	155	4.80%
Failure	Failure	1.4	120	4.30%
Anemia	Anemia	1.4	49	1.70%
Diarrhea	Diarrhea	1.5	44	1.60%
Tuberculosis (TB)	TB	1.4	38	1.40%

## Appendix A

**Table A1:** Summary of the techniques and methods.

Type of Data	Unit of Analysis	Analytical Methods	Presentations of Results
Abstracts Keywords	Words	Frequency of co-occurrence	1. The number of articles by countries mentioned in the abstract 2. Networks of co-occurrence authors’ keywords 3. Networks of countries by sharing co-authorships data
Abstracts	WoS research domains	Exploratory factor analysis	1. Top 50 research domains
Abstracts	Words	Jaccard’s similarity index	1. Top 50 most frequent co-occurrence terms
